# Detection of Lung Tumors in Mice Using a 1-Tesla Compact Magnetic Resonance Imaging System

**DOI:** 10.1371/journal.pone.0094945

**Published:** 2014-04-17

**Authors:** Fang Wang, Ken Akashi, Yoshinori Murakami, Yusuke Inoue, Toshihiro Furuta, Haruyasu Yamada, Kuni Ohtomo, Shigeru Kiryu

**Affiliations:** 1 Department of Radiology, Institute of Medical Science, University of Tokyo, Minato-ku, Tokyo, Japan; 2 Department of Radiology, Qi Lu Hospital of Shandong University, Jinan, China; 3 Division of Molecular Pathology, Institute of Medical Science, The University of Tokyo, Minato-ku, Tokyo, Japan; 4 Department of Diagnostic Radiology, Kitasato University School of Medicine, Minami-ku, Sagamihara, Kanagawa, Japan; 5 Department of Radiology, Graduate School of Medicine, University of Tokyo, Bunkyo-ku, Tokyo, Japan; Institute of Medical Science, University of Tokyo, Japan

## Abstract

Due to their small size, lung tumors in rodents are typically investigated using high-field magnetic resonance (MR) systems (4.7 T or higher) to achieve higher signal-to-noise ratios, although low-field MR systems are less sensitive to susceptibility artifacts caused by air in the lung. We investigated the feasibility of detecting lung tumors in living, freely breathing mice with a 1-T compact permanent magnet MR system. In total, 4 mice were used, and MR images of mouse lungs were acquired using a T1-weighted three-dimensional fast low-angle shot sequence without cardiac or respiratory gating. The delineation and size of lung tumors were assessed and compared with histopathological findings. Submillimeter lesions were demonstrated as hyperintense, relative to the surrounding lung parenchyma, and were delineated clearly. Among the 13 lesions validated in histopathological sections, 11 were detected in MR images; the MR detection rate was thus 84.6%. A strong correlation was obtained in size measurements between MR images and histological sections. Thus, a dedicated low-field MR system can be used to detect lung tumors in living mice noninvasively without gating.

## Introduction

Lung cancer is one of the most aggressive forms of malignant tumor, with a poor prognosis; it remains among the major causes of cancer-related death worldwide [1]. With more uniform and controlled conditions, experimental models are widely used for studying human diseases. Due to the similarities in terms of morphological, histogenic, and molecular characteristics, mouse models are a simple and orthotopic system for investigating human lung cancer [2]. Currently, available models include chemically induced models, transgenic models, and subcutaneous or orthotopic xenografts of human lung cancers, each of which have advantages and disadvantages [3]. Among them, chemically induced models are highly reproducible.

As one of the major imaging modalities, the main advantages of mouse lung CT are rapid data acquisition [4],[5] and inherently high contrast between the air and soft tissue. However, sufficient soft tissue contrast and spatial resolution depend on a high radiation dose, which may interfere with biological activity in the experimental animals and preclude repeated *in vivo* imaging [6].

As a non-invasive, versatile imaging modality, magnetic resonance (MR) imaging is commonly used to investigate small animals longitudinally *in vivo* [7]. Dedicated small animal MR scanners, with high spatial resolution and excellent soft tissue contrast, are now available commercially and have been used by many researchers [8],[9].

Several challenges exist for MR imaging of mouse lung, such as low proton density, susceptibility artifacts, sensitivity to respiratory and cardiac motion, relatively small size, and relatively long acquisition times [10]. However, the very factors, making it difficult to image normal lung, aid in the detection of tumors by increasing the contrast between normal and pathological tissue [7]. Although lung tumors of rodents are usually investigated using high-field superconducting systems (4.7 T or higher), a low-field system using a permanent magnet has been introduced for small animal imaging. Compared with high-field superconducting magnet MR imaging systems, advantages of the low-field permanent magnet MR imaging system include lower cost [11], improved contrast resolution, homogeneous RF fields [12], and reduced susceptibility effects [13]. Due to the poorer signal-to-noise ratio (SNR), compared with high-field MR imaging systems, there are few reports of the use of low-field MR imaging in assessing pulmonary tumors in living mice.

The aim of this study was to determine whether a low-field MR imaging system could be used routinely to identify and monitor the development of lung tumors in living mice. A mouse lung tumor model was induced and then the delineation of lung tumors was assessed using a 1-T permanent magnet MR imaging system in freely breathing mice.

## Materials and Methods

All animal experimental procedures were performed in accordance with the University of Tokyo guidelines for animal care and use, and approved by the Institute of medical science, the University of Tokyo animal care and use committee.

### Animals

This study was conducted using 4 female wild-type A/J mice (6 weeks old, SLC Japan, Hamamatsu, Japan). They were kept in specific pathogen-free conditions in an animal facility.

### Induction of animal model

As a major component of tobacco smoke, 4 (methylnitrosamino)-l-(3-pyridyl)-l-butanone (NNK) was used for the induction of lung tumor in mice [14]. NNK (Toronto Research Chemicals Inc., ON, Canada) was diluted with saline and mice were given a single intraperitoneal injection of NNK at a dose of 150 mg/kg at 6 weeks old to induce lung tumors. The corresponding study showed that proliferation of cells began to be detected 14 weeks after NNK treatment [14].

### MR imaging procedures

MR imaging was performed every 6 weeks, from 6 to 30 weeks after NNK treatment using the compact MR imaging system (MRmini; MRTechnology, Tsukuba, Japan), consisting of a console and permanent magnet of 1 T (Hitachi Metals, Tokyo, Japan). A solenoid coil of 30 mm inner diameter acted as the radiofrequency receiver coil. The specifications of the magnet were as follows: dimensions  = 60 (W)×64 (H)×82 (D) cm, weight  = 1400 kg, air gap  = 10 cm, and magnetic field homogeneity  = 3.6 ppm in a 30-mm diameter spherical volume [15].

Regular rodent pellet diet and water were available *ad libitum* until dietary preparation for MR imaging was started. The mice were then fed exclusively with autoclaved potatoes 24 h before MR imaging to reduce interference from gastrointestinal system signals [16]. Anesthesia was induced with 4% isoflurane and maintained with 1.5% isoflurane. When the breathing became regular, about 5 times every 10 seconds, mice were fixed on a polymethylmethacrylate holder in a prone position with adhesive tape, and put into the solenoid coil. Neither cardiac nor respiratory gating was used during data acquisition.

Scout images were acquired in the coronal plane in a T1-weighted three-dimensional fast low-angle shot (3D-FLASH) sequence with the following parameters: TR 30 ms, TE 2.2 ms, flip angle 51°, in-plane matrix 256×64, 50% rectangular field of view 3.3 cm, 8 slab partitions, number of excitations 1, bandwidth 100 kHz, and acquisition time 16 s. The voxel size was 0.26×0.52×2.08 mm. After the base of the left ventricle was set at the center of the magnet field, images were obtained in the coronal plane in T1-weighted 3D-FLASH with parameters: TR 30 ms, TE 2.2 ms, flip angle 64°, in-plane matrix 256×128, field of view 3.3 cm, 64 slab partitions, number of excitations 1, bandwidth 100 kHz, and acquisition time 4 min, 6 s. The voxel size was 0.26×0.26×0.52 mm. Using zero interpolation, we obtained 0.13-mm isotropic voxels with no interslice gaps.

### Histological examination

At the final imaging time point, during week 30 after carcinogen exposure (36 weeks old), mice were scanned and then sacrificed immediately for histological analyses. The lungs were cut coronally. Tissue sections were stained with hematoxylin and eosin and examined by light microscopy. Histological results of lesions were used as the “gold standard” to assess the MR images.

### Image analysis

Image analyses were performed using the ImageJ software (US National Institutes of Health, Bethesda, MD). Reformatted images in the transverse plane were produced using the ImageJ software. Two radiologists examined MR images of four mice from different time points independently. The lesions were identified in both the coronal and transverse images as increased signal intensities relative to the surrounding normal lung tissue in two or more contiguous slices and were monitored according to their locations.

For descriptive purposes, in coronal images, each lung was divided into upper and lower lung fields, on the central axial image at the middle level between the apex and the lowest point of the lung. Once lesions were observed, observers recorded the location of each lesion and number of lesions throughout the whole lung for each mouse. Consensus between observers was reached through discussion. The longest diameter of each lesion was assigned as the lesion size and measured manually in histological sections and coronal MR images obtained on the same day. The Pearson product-moment correlation test was used for correlation analyses of lesion size between MR images and histological sections. All statistical analyses were performed using EZR (Saitama Medical Center, Jichi Medical University).

## Results

Although slight blurring of the diaphragm was observed in some cases, no severe motion-related artifact was observed before tumors developed. The image quality was adequate to detect and evaluate lung lesions. Pulmonary vessels were clearly imaged as high signal intensities, with the lung parenchyma as a dark background area on T1-weighted FLASH images.

**Figure 1:**
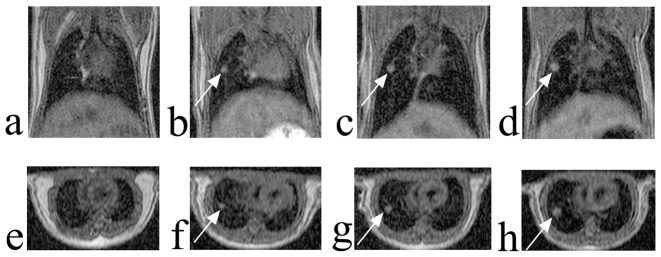
Longitudinal MR images of a lung tumor in an individual mouse. No lesion was detected at 12 weeks after NNK treatment (a). At 18 weeks, a small lesion was identified (b). This lung tumor increased in size over time, at 24 (c) and 30 weeks (d). The lower panels (e, f, g, h) are the corresponding axial reformatted images for a, b, c, and d, respectively. The lesion was found to be an adenoma by histological examination.

At the initial two MR scans, performed 6 and 12 weeks after NNK treatment, no lesion was seen (Fig 1). At 18 weeks after treatment, lesions became detectable; three lesions in three mice were seen. Lesions were demonstrated as hyperintense, relative to surrounding lung parenchyma, and were well-defined. Among them, the smallest lesion detected was about 0.5 mm in diameter, comparable with a literature report [10], [17]. The time course of lesion growth was successfully observed by serial MR imaging (Fig. 1).

**Figure 2:**
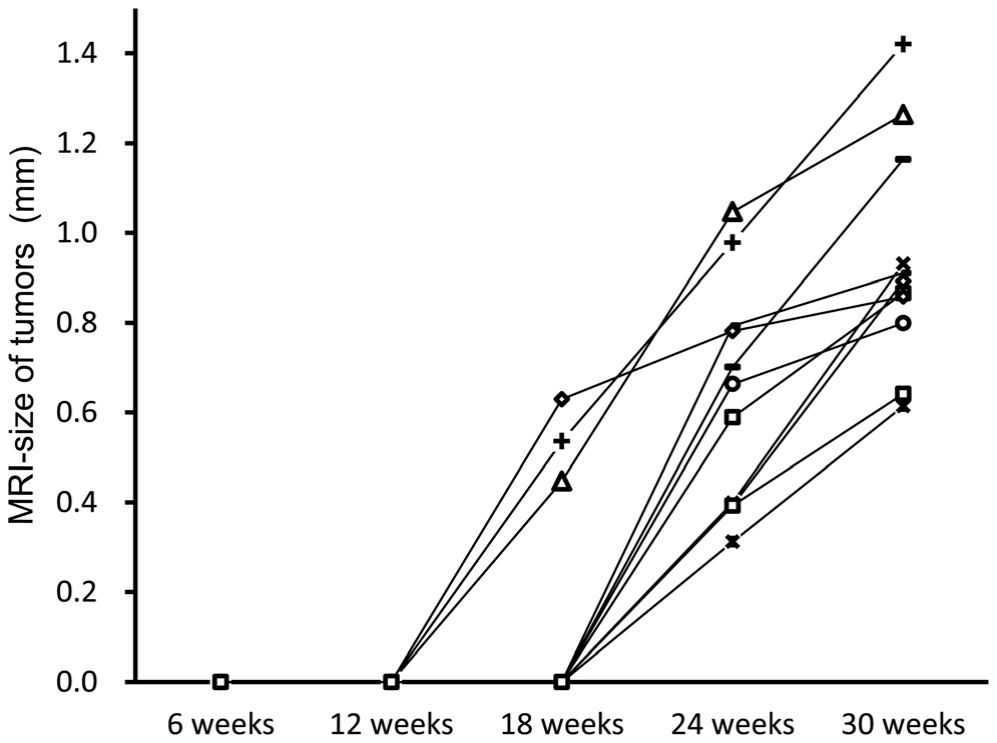
The MRI size of each lesion at each time point. Three lesions were detected at 18 weeks and grew in size over the following time. A total of 11 lesions was detected at 24 weeks and kept growing in size until 30 weeks, the end of the study.

At 24 weeks after treatment, 11 discrete nodular lesions were detected in four mice. These masses showed slight increases in size, but not in number, at 30 weeks after administration of NNK. Lesions were distributed as follows: two in the left upper lung field, three in the left lower lung field, two in the right upper lung field, and four in the right lower lung field. The MRI size of each lesion at each time point is shown in Fig. 2. All detected lesions grew in size longitudinally, and none of them decreased in size or disappeared later.

In total, 13 nodular lesions were detected histologically, with two or more lesions in each mouse. Histological examination showed that the most common lesion was an adenoma: 9 of 13 lesions. Two carcinomas were observed in two mice. A case of arteritis was found in one mouse. One lesion was frozen for a further analysis, and no pathological result was obtained for this lesion.

**Figure 3:**
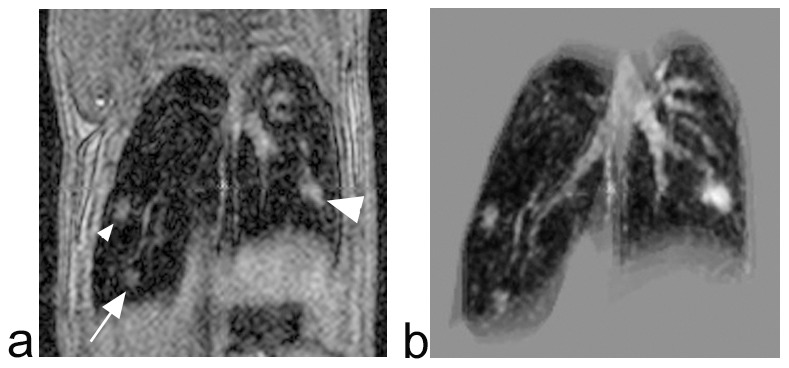
Representative coronal T1-weighted MR images of lung tumors. Two lung tumors located in the right lung (small arrowhead and arrow) and one lung tumor located in the left lung (large arrowhead) (a). Three-dimensional volume-rendered image generated from the 3D data showing the relative locations of tumors and pulmonary vessels (b).

**Figure 4:**
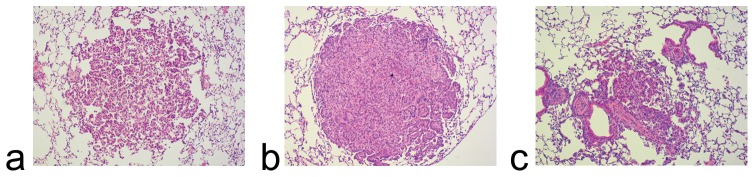
Hematoxylin and eosin staining of lung lesions. Panels a, b, and c show histological images of the lung lesions in Figure 2, indicated by the small arrowhead, long arrow, and large arrowhead, respectively. The lesions were found to be adenomas (a and c) and a carcinoma (b) by histological examination.

All lesions identified in MR images were also demonstrated in histological sections, with well-matched locations between them (Figs. 3,4). MR images revealed 11 of 13 (84.6%) lesions detected in histological sections. Two lesions in two mice identified by histology were not detected in MR images: one connected with the right diaphragm, histologically validated as arteritis, and the other, adjoining the left hilum, identified as a carcinoma.

**Figure 5:**
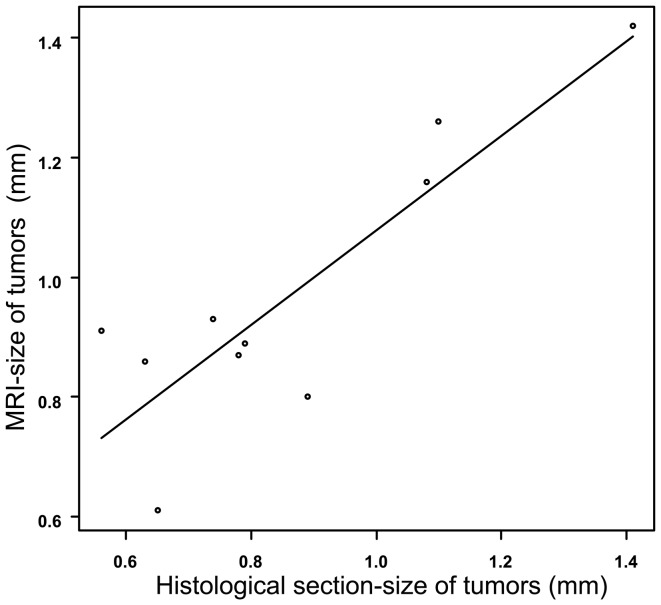
Correlation of tumor size determined by MR imaging and histological evaluation (correlation coefficient  = 0.87).

The lesions ranged from 0.61 to 1.54 mm in diameter, with average size of 1.05±0.29 mm (mean±SD), in final MR images and from 0.56 to 1.41 mm in diameter, with an average size of 0.87±0.26 mm, in histological sections. A significant correlation coefficient of 0.87 (P = 0.001) was obtained between lesion detection by MR imaging and histological evaluation (Fig. 5).

## Discussion

In this research, we delineated lung nodular lesions using a 1-T compact permanent magnet MR system. In this study, submillimeter lung lesions were induced by treatment with NNK and MRI could delineate these lesions at a detection rate of 84.6%. MRI also allows observation of the time course of tumor growth. A strong correlation was obtained in measurements of tumore size by MR imaging and histological evaluation.

To date, most studies of lung tumors in rodents have been performed using higher-field MR, with static field strengths of 4.7 T [7]–[10],[17] – [22] or higher [23] – [25]. Compared with high-field MR, the main disadvantage of low-field MR is the reduced SNR [13]. To achieve a higher SNR, we used the shortest TE for this 1-T MR system. The low proton density in the surrounding lung parenchyma may contribute to lesion detection with the 1-T MR system. Also, low-field MR scanners suffer from fewer susceptibility artifacts, which is important in lung imaging. With low-field MR scanners, we seldom encounter other field-dependent image artifacts or reduced image contrast, which often occur at higher field strengths [26],[27]. Also, low-field MR can be a cost-effective alternative to higher-field MR for a number of research applications [11].

3D-FLASH is a rapid imaging sequence, with optimized SNR, and represents a variable compromise between SNR and temporal and spatial resolution. It is suitable for small animal imaging. Compared with a spin-echo sequence, a T1-weighted 3D-FLASH sequence can produce thinner MR images [15]. Although the in-plane resolution of a T1-weighted 3D-FLASH sequence is not higher than a spin-echo sequence, thinner MR images enables isotropic acquisition. Regardless of the orientation of the original acquisition, isotropic voxels of 3D-FLASH can provide reformatted images of high spatial resolution in any arbitrary plane. The combination of two planes facilitates the identification of tumors with excellent anatomical information. In this study, we acquired original images of tumors in the coronal plane using 3D-FLASH at high spatial resolution, and reformatted images in the transverse plane. In total, two tumors in two mice identified by histology were missed in MR images, presumably due to continuity of the lesions with surrounding structures, one in contact with the right diaphragm, and the other abutting left lung hilar structures. MR detection of tumors located near or in contact with the heart, thick vessels, and diaphragm appears to be difficult, especially in the case of tiny lesions, partly due to mild motion-related blurring. The generally lower contrast of 3D-FLASH may also be a factor [15].

For MR images without gating, the degree of motion artifact is determined by the number of motion events and the magnitude of the motion in the imaging volume [28]. In previous research assessing lung tumors, most researchers applied respiratory gating [7],[18],[19],[22],[24], some used both respiratory and cardiac gating [8],[9],[17],[20],[21],[23], and acceptable image quality was generally achieved. In this study, neither respiratory nor cardiac gating was applied, however, minor or modest artifacts, acceptable for routine, did not impair image evaluation and MRI is capable of showing submillimeter lesions.

The following factors may have helped to minimize artifacts. First, low-field MR system was used in this study. In higher-field MR system, SNRs increased the conspicuity of ghost which is produced by breathing [29]. Therefore, breathing artifacts would be reduced at low field strengths. The weak susceptibility effects of low-field MR system may also contribute to the detection of lung lesions. Second, during the scan, mice were fixed on a holder, with tape closely fitted around the chest, which helped to restrain respiratory motion [15]. Using this technique, tape fastening should not be too tight not to restrict normal breathing of mice and to minimize animal suffering consistent with animal ethics. Omission of gating makes MR imaging more convenient and is able to achieve reasonable acquisition time, nevertheless, we must always remember higher SNR can be obtained by using gating system.

With higher water content, tumors were demonstrated as hyperintense areas, relative to the surrounding lung parenchyma, isointense to adjacent soft tissues, such as the heart. MRI can show the tumor's location, shape, and size. In this study, image quality was adequate to detect and measure lung tumors. In MR imaging, spatial resolution is mainly determined by slice thickness, matrix size, and the field of view. The smallest lesion detected in this study was 0.5 mm in diameter, as in higher-field MRI [10],[17]. Kubo et al. assessed MR imaging of lung tumors using a 4.7 Tesla superconducting MR system and found that abnormalities were detected with a more than 96% histopathological correlation [20]. In their research, other lung abnormalities, such as atelectasis, pleural thickening and etc., were also evaluated besides lung tumor. In the present study, our concern was concentrated on the detection of lung nodular lesion and the detection rate was 84.6%. Some differences are expected to exist between MR systems with higher magnetic field or lower magnetic field, and still, a similar result was obtained using a lower field MR system without gating system.

Except two nodules not visualized on MR imaging, the strong correlation of tumor sizes determined by MR imaging and histological evaluation. This indicates that MR imaging permits noninvasive, longitudinal monitoring of lung tumor progression. Compared with histological sections, MR images slightly overestimated the size of tumors, which may be attributable to both an integrated volume resulting from respiratory motion of the lung tumors and tumor shrinkage from dehydration [30]. In this paper, the time course of tumor progression was observed and the growth rate was also attained using a 1-T compact MR scanner. Thus, there is a reason to expect that the feasibility of 1-T compact MRI to monitor the effect of therapy of lung tumor.

## Conclusions

In conclusion, our initial results demonstrated the feasibility of detecting lung nodular lesions in living mice using a 1-T compact MR system without respiratory or cardiac gating. With reasonable scan times and image quality, this will be a valuable tool for monitoring tumor progression and evaluating responses to treatment.
